# Chronic diseases, inflammation, and spices: how are they linked?

**DOI:** 10.1186/s12967-018-1381-2

**Published:** 2018-01-25

**Authors:** Ajaikumar B. Kunnumakkara, Bethsebie L. Sailo, Kishore Banik, Choudhary Harsha, Sahdeo Prasad, Subash Chandra Gupta, Alok Chandra Bharti, Bharat B. Aggarwal

**Affiliations:** 1 Cancer Biology Laboratory and DBT-AIST International Laboratory for Advanced Biomedicine (DAILAB), Department of Biosciences and Bioengineering, Indian Institute of Technology Guwahati, Guwahati, Assam 781039 India; 2 University of Texas MD Anderson Cancer Center, Houston, TX USA; 3 Department of Biochemistry, Institute of Science, Banaras Hindu University, Varanasi, 221005 India; 4 Molecular Oncology Laboratory, Department of Zoology, University of Delhi (North Campus), Delhi, 110007 India; 5 Inflammation Research Center, San Diego, CA USA

**Keywords:** Spices, Chronic diseases, Inflammation, Cancer, NF-κB, STAT3

## Abstract

Extensive research within the last several decades has revealed that the major risk factors for most chronic diseases are infections, obesity, alcohol, tobacco, radiation, environmental pollutants, and diet. It is now well established that these factors induce chronic diseases through induction of inflammation. However, inflammation could be either acute or chronic. Acute inflammation persists for a short duration and is the host defense against infections and allergens, whereas the chronic inflammation persists for a long time and leads to many chronic diseases including cancer, cardiovascular diseases, neurodegenerative diseases, respiratory diseases, etc. Numerous lines of evidence suggest that the aforementioned risk factors induced cancer through chronic inflammation. First, transcription factors NF-κB and STAT3 that regulate expression of inflammatory gene products, have been found to be constitutively active in most cancers; second, chronic inflammation such as pancreatitis, prostatitis, hepatitis etc. leads to cancers; third, activation of NF-κB and STAT3 leads to cancer cell proliferation, survival, invasion, angiogenesis and metastasis; fourth, activation of NF-κB and STAT3 leads to resistance to chemotherapy and radiation, and hypoxia and acidic conditions activate these transcription factors. Therefore, targeting these pathways may provide opportunities for both prevention and treatment of cancer and other chronic diseases. We will discuss in this review the potential of various dietary agents such as spices and its components in the suppression of inflammatory pathways and their roles in the prevention and therapy of cancer and other chronic diseases. In fact, epidemiological studies do indicate that cancer incidence in countries such as India where spices are consumed daily is much lower (94/100,000) than those where spices are not consumed such as United States (318/100,000), suggesting the potential role of spices in cancer prevention.

## Background

Chronic diseases, also called as non-communicable diseases that include Alzheimer’s disease, arthritis, cancer, cardiovascular disease (CVD), diabetes and Parkinson’s disease, remain the primary root cause of death and disability worldwide [1–3]. The major risk factors associated with these diseases are unhealthy lifestyle including lack of physical activity, poor diet, stress, excessive tobacco and alcohol consumption, exposure to radiation, and infection with pathogenic microorganisms. It is now well established that these agents induce inflammation and dysregulate inflammatory pathways, which lead to the development of chronic diseases [1–3].

Inflammation, which means, “to set on fire” is a body’s natural response against harmful pathogen and stimuli that occurs in two stages namely, acute and chronic inflammation [4]. Acute inflammation is a part of innate immunity initiated by the immune cells that persists only for a short time. However, if the inflammation continues, the second stage of inflammation called chronic inflammation commences which instigates various kinds of chronic diseases, including arthritis, cancer, cardiovascular diseases, diabetes, and neurological diseases via dysregulation of various signaling pathways such as nuclear factor kappa-B (NF-κB), signal transducer and activator of transcription 3 (STAT3) etc. [5]. Hence, targeting the inflammatory pathways has high potential in preventing and eradicating these deadly diseases [1]. However, most of the drugs developed till today for the treatment of chronic diseases are highly expensive and associated with adverse side effects [1]. Therefore, there is an urgent need to develop novel, safe, affordable, and highly efficacious agents for the management of these diseases.

Congregate evidence suggests that a diet rich in plant-based agents including spices has the ability to prevent most of the chronic diseases. The earliest evidence of the use of spices by humans dates back to 5000 B.C., and till today their biological activities have been extensively studied [6]. “Spice” originates from the Latin word, “*species”*, which means a commodity of special distinction or value [7]. Spices have been extensively used since ancient times as means of remedy, coloring agent, flavoring agent, and preservative. Subsequently, tremendous studies have shown that nutraceuticals derived from spices such as clove, coriander, garlic, ginger, onion, pepper, turmeric, etc., remarkably prevent and cure various chronic diseases by targeting inflammatory pathways [8]. This review emphasizes the association between inflammation and chronic diseases and the benefits of spices in warding off these global major health issues.

## Molecular pathways linked to inflammation

**Fig. 1:**
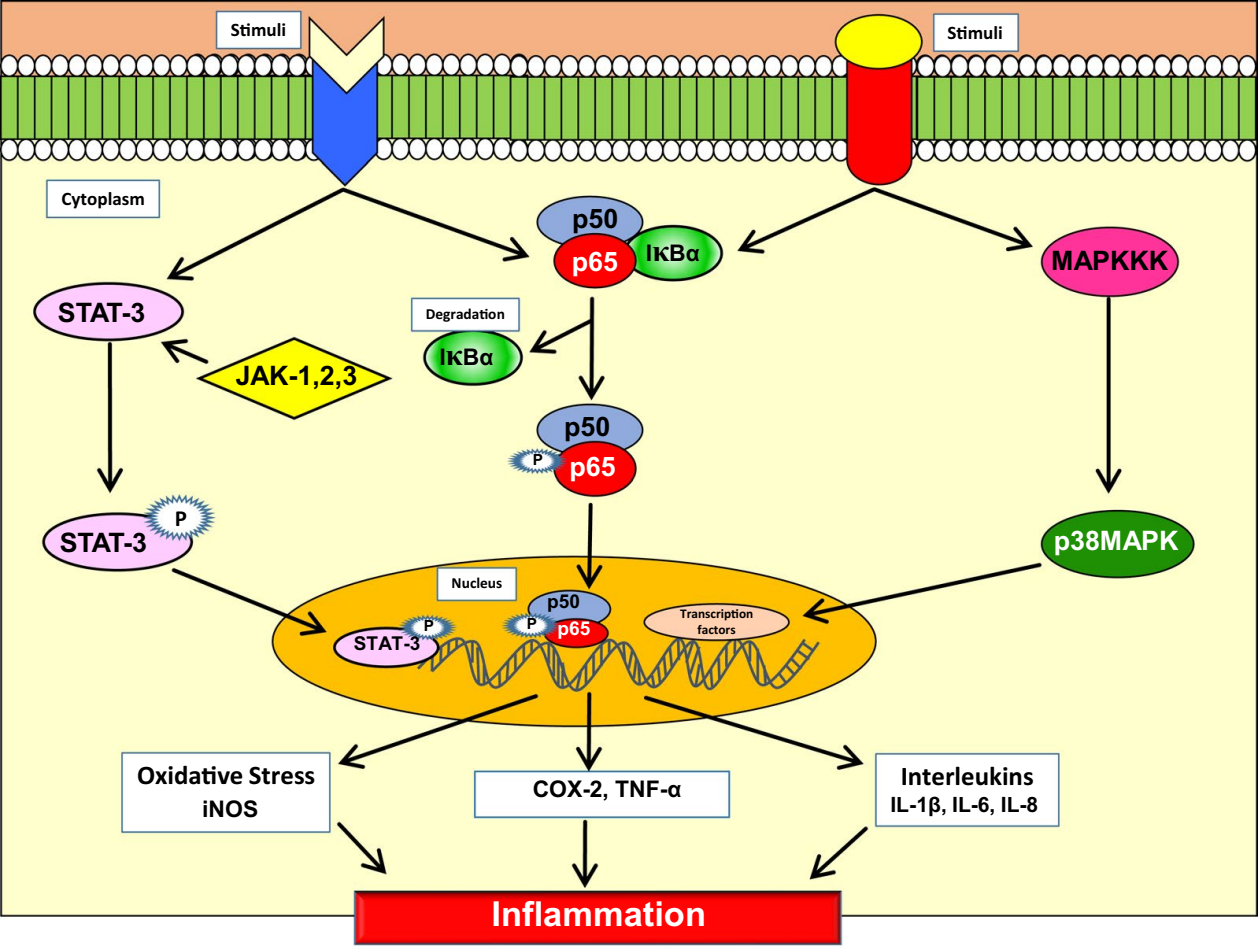
Molecular pathway of inflammation linked to chronic diseases

Aforementioned, inflammation is essentially an immune response to infection or injury in the body that helps to maintain tissue homeostasis under stressful conditions [9]. Eventually, it was discovered that transcription factors such as NF-κB and STAT3, inflammatory enzymes such as cyclooxygenase-2 (COX-2), matrix metalloproteinase-9 (MMP-9), and inflammatory cytokines such as tumor necrosis factor alpha (TNF-α), interleukins (IL) such as IL-1, -6, -8, and chemokines are the main molecular mediators of this response. Amongst these mediators, ubiquitous transcription factor NF-κB is the key mediator of inflammation as it regulates large arrays of genes encoding cytokines, cytokine receptors, and cell adhesion molecules that are involved in triggering inflammation [10, 11]. In normal condition, NF-κB exists in the cytoplasm in the form of a heterotrimer that comprises of the subunit p50, p65, and inhibitory subunit IκBα. Upon activation by certain inflammatory stimuli, cytokines, carcinogens, free radicals, tumor promoters, UV-light, γ-rays, and x-rays, the subunits p50 and p65 translocate into the nucleus, bind to the promoters region of various genes, and activate more than 400 genes that are involved in inflammation and other chronic diseases [12] (Fig. 1). Activation of NF-κB is also known to instigate cancer cell proliferation, survival, invasion, angiogenesis, metastasis, chemoresistance, and radiation resistance.

NF-κB regulates the expression of inflammatory mediators such as COX-2, inducible nitric oxide synthase (iNOS), TNF-α, and interleukins [11]. Overexpression of the cytokine, TNF-α, the most potent pro-inflammatory cytokine so far discovered, can lead to various chronic diseases, including cancer, via the activation of NF-κB. Therefore, the blockers of TNF-α have high potential for the prevention and management of chronic diseases and the global market for TNF-α blockers is approximately $20 billion. However, most of these blockers that have been approved for the treatment of chronic diseases are very expensive and have numerous adverse side effects. Interleukins are a group of cytokines that are released by macrophages. Interleukins such as IL-1β, IL-6 and IL-8 also play pivotal roles in inducing inflammatory response [10]. Upregulation of COX-2, iNOS, and aberrant expression of TNF-α and IL-1, IL-6 and IL-8 have been reported to play important roles in oxidative stress that leads to inflammation [5].

IL-6 is a key NF-*κ*B-dependent cytokine that induces the activation of STAT3. STAT3 is a cytoplasmic protein that acts as a transcriptional factor and induces several types of immune and inflammatory responses. The activation of STAT3 involves tyrosine phosphorylation, homodimerization, nuclear translocation where it binds to the DNA and regulates gene transcription [6, 13] (Fig. 1). Protein kinases such as Janus-activated kinase (JAK) 1, 2, and 3 were found to phosphorylate STAT3 and induce its nuclear translocation [6].

Besides these, other transcription factors such as activator protein-1 (AP-1), hypoxia-inducible factor-1α (HIF-1α), nuclear factor of activated T cells (NFAT) and nuclear factor erythroid 2–related factor 2 (Nrf2) are also modulated by inflammatory cytokines and play crucial function for mediating cellular stress responses [5]. The mitogen-activated protein kinase (MAPK) family consisting of three different stress-activated protein kinase pathways namely p38, JNK and ERK, has been found to modulate the level of IL-5 and other cytokines during inflammation. Therefore, MAPK pathway can also be used as a potential molecular target for the treatment of chronic inflammatory diseases [14] (Fig. 1).

## Chronic diseases and inflammation

Chronic diseases are the leading cause of mortality in the world accounting for approximately 60% of all deaths. Aforementioned, various inflammatory biomarkers are altered in chronic diseases such as transcription factors (NF-κB, STAT3) and their downstream products such as inflammatory cytokines (TNF-α, IL-1, IL-6, IL-8) and pro-inflammatory enzymes such as COX-2, MMP-9, cell adhesion molecules (CAM), vascular endothelial growth factor (VEGF) etc. [1, 15].

Amongst the chronic diseases, cancer is one of the major diseases caused by chronic inflammation. In 2009, Colotta et al. proposed inflammation as the seventh hallmark of cancer [16]. Both inflammation and cancer are linked through intrinsic and extrinsic pathways i.e. oncogenes regulate the inflammatory microenvironment intrinsically, whilst the inflammatory microenvironment facilitates the development and progression of cancer extrinsically [17]. Specifically, the inflammatory response positively aids in tumor development and increases the risk of malignancy [18]. Approximately 15% of the cancer cases are caused by persistent infection and chronic inflammation [19]. It has been well established that NF-κB is constitutively activated in various cancers such as cancers of the breast, colon, liver, lung, pancreas etc. in response to carcinogens such as tobacco, alcohol, and exposure to radiation etc. Upregulation of NF-κB subsequently activates hundreds of pro-inflammatory gene products including TNF-α, IL-1, IL-6, chemokines, MMP-9, 5-LOX, VEGF, and COX-2 [20]. These pro-inflammatory cytokines play a vital role in inflammation-induced cancer cell proliferation, angiogenesis, invasion, metastasis, and suppression of apoptosis. In addition, even in cancers that are not instigated by inflammation, inflammatory cells enter the tumor stroma and consequently induce cancer development [21]. More importantly, an in vivo study has illustrated that NF-κB activation via the IκB kinase (IKK) complex acts as a molecular link between inflammation and cancer [22]. Moreover, NF-κB activation also leads to radioresistance and chemoresistance. These observations suggest that NF-κB plays an important role in inflammation and cancer. Therefore, anti-inflammatory agents that target NF-κB and its regulated products may have high efficacy in both the prevention and treatment of cancers.

Inflammatory cytokines IL-1 and IL-6 also modulate pro-oncogenic transcription factor STAT3, thereby increasing survival, proliferation, angiogenesis, invasion, and metastasis of cancer cells [23]. STAT3 was also known to be upregulated in many cancer patients, and the level of STAT3 was directly correlated with poor prognosis [1]. In case of oral cancer, oral submucous fibrosis or oral lichen planus are precancerous conditions implicated with immuno-inflammatory processes that may transform to cancer [24]. Besides, chronic inflammation in various organs or tissues leads to different types of cancers. For example, chronic obstructive pulmonary disease (COPD) leads to lung cancer, colitis leads to colon cancer, gastritis leads to stomach cancer, pancreatitis leads to pancreatic cancer, prostatitis leads to prostate cancer, etc. [25–28].

Aforesaid, unresolved inflammation of the pancreas, pancreatitis leads to pancreatic cancer. It has been demonstrated that O-GlcNAc transferase (OGT)—mediated O-GlcNAcylation activated NF-κB signaling pathway and inflammation in pancreatic acinar cells, ultimately leading to the progression of acute pancreatitis [29]. T helper cell-mediated inflammation also has been found to be associated with pancreatic β-cell dysfunction and leads to chronic pancreatitis [30]. COPD is an epidemic chronic inflammatory disease of the lung [31, 32]. Interleukin-33 enhances the production of the inflammatory cytokine such as IL-6 and IL-8 in chronic airway inflammation, thus contributing to COPD development [33]. It has also been reported that inflammatory responses in COPD promote lung tumor initiation and progression [34]. Another inflammation induced chronic disease is rheumatoid arthritis (RA) which is an autoimmune disease characterized by the production of the pro-inflammatory cytokine IL-17 [35]. Studies suggested that pro-inflammatory cytokines such as IL-1β, IL-6 and TNF-α also play pathological roles in the development of RA [36]. In addition, it has been demonstrated that STAT3 also caused chronic inflammation and joint destruction in RA [36]. Hence, targeting inflammatory pathways can be used for the prevention and treatment of RA.

In Alzheimer’s disease (AD), which is the prevalent chronic neurodegenerative disease, inflammation has an essential role in the disease pathogenesis. Studies have indicated that microRNAs, astrocytes, microglia, and infiltrating immune cells from the peripheral region might affect the development of neuroinflammation and neurodegeneration in AD patients [37]. Accumulated evidence has depicted that deposition of extracellular amyloid beta (Aβ) in AD leads to upregulation of pro-inflammatory mediators IL-1β, IL-6 and TNF-α, by the activated immune cells, which promote additional inflammatory pathways via instigation of COX-2 and NF-κB [37].

Inflammatory bowel disease (IBD) is a group of inflammatory disorders of the digestive tract, which mainly includes Crohn’s disease and ulcerative colitis. Studies have shown that IBD patients have high susceptibility to develop colorectal cancer. Inflammatory mediators including cytokines (TNF-α, IL-1β, IL-6, IL-17, and IL-21), eicosanoids, and reactive oxygen metabolites play a vital role in causing the chronic inflammatory condition in IBD [13, 38]. In addition, activation of STAT3 signaling pathway is associated with colitis and colorectal cancer [39].

Allergic asthma is an airway inflammatory disease caused due to exposure to allergens causing bronchoconstriction. Asthma is characterized by an imbalance between the T helper type 1 (Th1) and T helper type 2 (Th2) responses and excessive production of reactive oxygen species (ROS) [40]. Th2 cells release several cytokines such as IL-4 and IL-13 that in turn produces immunoglobulin, IgE resulting in allergic response [41]. Numerous studies also indicate that attenuation of the Type 2 inflammatory pathway caused a clinically substantial reduction in asthma exacerbations. Thus, it is now evident that type 2 inflammation is an imperative mechanism of susceptibility to asthma exacerbation [42].

Diabetes mellitus (DM) is a predominant metabolic chronic disease that affects more than 170 million people globally. Type 1 DM is induced by the chronic inflammation of pancreatic islets, while type 2 DM is associated with insulin resistance resulting in elevated production of inflammatory markers such as C-reactive protein (CRP), IL-6, and TNF-α [43]. Patients with type 2 diabetes have a higher chance of developing atherosclerosis, which is a disease wherein plaque accumulates in arteries. Arachidonic acid derived eicosanoids such as prostaglandin E_2_ (PGE_2_) and leukotriene B4 (LTB4) are the potential pro-inflammatory mediators in atherosclerosis and are regulated by NF-κB [43].

Collectively, it is apparent that dysregulation of inflammatory pathways is the underlying mechanism of various chronic diseases. Therefore, many drugs have been developed that target inflammatory pathways for the management of these diseases. However, most of these drugs developed so far are highly expensive and are not devoid of adverse side effects. Hence, there is an urgent need to develop safe, affordable, and efficacious drugs for the prevention and treatment of these chronic diseases. It has been well established that the population who consume spices are less susceptible to the development of chronic diseases. The components present in these spices have the ability to inhibit inflammatory pathways that lead to chronic inflammation, which contributes to the biological properties of these spices.

## Spices and their active components

Mother nature has bestowed us with a profuse source of remedies to treat various kinds of ailments. Since time immemorial, phytochemicals, both in their natural as well as synthetic forms have been used for the treatment of various chronic diseases [12]. The root, leaf, bud, seed, bark, berry, stigma of a plant or flower used for the culinary purpose are generally called as spices. Spices not only add flavor and taste to food, but also exhibit tremendous health benefits [44]. Numerous results from preclinical and clinical studies over the past several decades have ascertained the efficacious role of spices and their active components in preventing and combating various diseases including arthritis, asthma, cancer, cardiovascular diseases, diabetes, and neurodegenerative diseases [45]. The most commonly used spices for culinary purpose that shows biological activities are black pepper, cardamom, cinnamon, clove, cumin, fenugreek, fennel, garlic, ginger, onion, rosemary, turmeric etc.

**Fig. 2:**
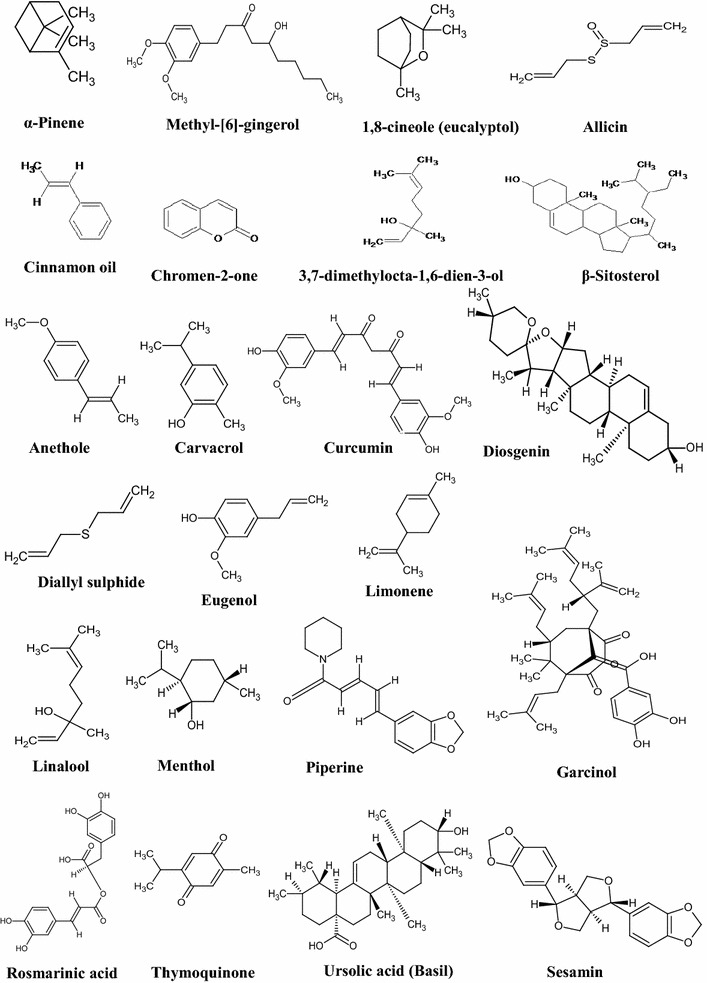
Structures of active components of spices

Turmeric (*Curcuma longa*) is the most commonly used spice in the world. Curcumin, the main component of turmeric (2–5%), obtained from rhizomes of this plant, is a yellow colored compound, which gives the golden color to turmeric, was first isolated by Vogel in 1842. In 1910, the structure of curcumin was determined as diferuloylmethane and later synthesized and cocrystallized with 5-LOX in 2003 [46]. This ‘golden spice’ is recognized for its anti-inflammatory, antimicrobial, insecticidal, antimutagenic, radioprotective, and anticancer properties. Over ten thousand studies have been reported in the literature about the biological activities of this compound including more than 120 clinical trials. Besides curcumin, the other active components of turmeric include demethoxycurcumin, bisdemethoxycurcumin, sesquiterpenes, diterpenes, triterpenoids, [47, 48]. Black pepper (*Piper nigrum)*, another commonly used spice is widely known for its immunomodulatory, anti-oxidant, anti-asthmatic, anti-carcinogenic, anti-inflammatory and anti-ulcer properties [49]. Other than its main component piperine, black pepper also contains β-caryophyllene, limonene, δ-3-carene, α-pinene, β-pinene, α-phellandrene, myrcene, terpinolene, etc. [50]. Another extensively used spice, ginger (*Zingiber officinale*) is reported to have different biological properties such as antioxidant, anti-inflammatory and antiproliferative properties. 6-gingerol is the main component of this spice, which is responsible for its biological properties [51]. Other than gingerol, ginger also contains 6-paradol, 6-gingerdiol, gingerdione, shogoal, zingiberene, citral (neral and geranial), bisabolene, cineol, α-farnesene, β-phellandrene, zingerone etc. [52]. The most commonly used spice for cardiovascular diseases in the ancient system of medicine is garlic (*Allium sativum*). It also possesses anti-inflammatory, gastroprotective and anti-cancer properties due to the presence of phytochemicals such as diallyl sulfides, diallyl disulfides, ajoene, allicin, alliin, diallyl trisulfide, S-allylcysteine, methiin, isoalliin, cycloalliin, S-allylmercaptocysteine [53, 54]. Another spice that is widely used all over the world to enhance the spice level of dishes is red pepper (*Capsicum*). Apart from capsaicin, red pepper also contains β-carotene, zeaxanthin, lutein, caffeic acid and capsanthin [55]. The other commonly used spices and their active components include cardamom (1,8-cineole, α-terpinyl acetate, limonene, linalool, linalyl acetate, terpinolene and myrcene) [4, 56]; cinnamon (cinnamaldehyde, cinnamyl acetate, cineole, coumarin, ethyl cinnamate, linalool, humulene, β-caryophyllene, τ-cadinol) [57, 58]; clove (eugenol) [4]; fenugreek (diosgenin, yamogenin, choline, resins, trigonelline) [59]; black cumin (thymoquinone, cuminaldehyde, γ-terpinene, β-pinene, *p*-mentha-1, 3-diene-7-al, *p*-mentha-1, 4-dien-7-al, *p*-cymene) [60]; kokum (garcinol, xanthochymol, isoxanthochymol, 1,2-dihydroxypropane-1,2,3-tricarboxylic acid) [61]; rosemary [bornyl acetate, rosmarinic acid, carnosol, carnosic acid, camphor, limonene, camphene, borneol, cineole, α-pinene, (Z)-linalool oxide] [62]; saffron (crocetin and crocin) [63]; star anise (estragole, trans-anethole, limonene) etc. [64]. Hence, it is evident that spices contain a diverse range of active components that provide tremendous health benefits. Table 1 shows a list of spices, their common names, scientific names, and their active components. Figure 2 depicts the structures of active components of spices.

**Table 1:** Spices and their major components

Spice	Scientific name	Major components	References
Anise	*Pimpinella anisum*	Anethole, estragole, γ-hymachalen, para-anisaldehyde, methyl cavicol	[164]
Asafoetida	*Ferula asafetida*	Ferulic acid, umbel-liferone, asaresinotannols, farnesiferols A, B, C, glucose, galactose, l-arabinose, rhamnose, glucuronic acid, 2-butyl propenyl disulfide	[165]
Basil	*Ocimum basilicum*	Estragole, linalool, 1, 8-cineole, eugenol, methyl cinnamate, α-cubebene, α-farnesene, caryophyllene, β-ocimene	[166]
Bay leaves	*Laurus nobilis*	1,8-cineole, α-pinene, limonene, alpha-terpinyl acetate, terpinene-4-ol	[167, 168]
Black cumin	*Nigella sativa*	Thymoquinone, cuminaldehyde, γ-terpinene, β-pinene, *p*-cymene, *p*-mentha-1,3-diene-7-al, *p*-mentha-1,4-dien-7-al	[60, 169]
Black pepper	*Piper nigrum*	Piperine, β-caryophyllene, limonene, δ-3-carene, α-pinene, β-pinene, α-phellandrene, myrcene, terpinolene	[50]
Cardamom	*Elettaria cardamomum*	1,8-cineole, α-terpinyl acetate, limonene, linalool, terpinolene, myrcene, linalyl acetate	[56]
Celery seed	*Trachyspermum ammi*	2 Isopropyl-5-methyl-phenol, octadecanoic acid, lupeol acetate, hexadecanoic acid, (3β, 24S)-stigmast-5-en-3-ol, stigmasta-5,22-dien-3β-ol, lup-20(29)-en-3-yl acetate	[170]
Cinnamon	*Cinnamomum zeylanicum*	Cinnamaldehyde, cinnamyl acetate, cineole, eugenol, coumarin, linalool, humulene, ethyl cinnamate, β-caryophyllene, τ-cadinol	[58]
Clove	*Syzygium aromaticm*	Eugenol, eugenyl acetate, α-humulene, β-caryophyllene	[171]
Coriander	*Corriandrum sativum*	Petroselinic acid, linoleic acid, oleic acid, palmitic acid, stearic acid, vaccenic acid, myristic acid	[172]
Dill	*Anethum graveolens*	α-Phellandrene, limonene, dill ether, sabinene, α-pinene, *n*-tetracosane, neophytadiene, *n*-docosane, *n*-tricosane, *n*-nonadecane, *n*-eicosane, *n*-heneicosane, β-myrcene, α-tujene	[173]
Fennel	*Foeniculum vulgare*	Estragole, trans-anethole, fenchone, limonene, anisaldehyde, sabinene, β-myrcene, α-pinene, β-pinene, camphene	[174]
Fenugreek	*Trigonella foenum-graecum*	Diosgenin, yamogenin, gitogenin, tigogenin, neotigogens, carpaine, trigonelline, gentianine, 4-hydroxyisoleucine, fenugreekine, choline	[59]
Garlic	*Allium sativum*	Diallyl sulfides, diallyl disulfides, diallyl trisulfide, ajoene, allicin, alliin, methiin, S-allylcysteine, isoalliin, cycloalliin, S-allylmercaptocysteine	[51]
Ginger	*Zingiber officinale*	[6]-gingerol, [6]-paradol, shogoal, 6-gingerdiol, gingerdione, zingiberene, citral (neral and geranial), bisabolene, α-farnesene, β-phellandrene, cineole, zingerone	[52, 175]
Kokum	*Garcinia indica*	Garcinol, xanthochymol, isoxanthochymol, 1,2-dihydroxypropane-1,2,3-tricarboxylic acid	[61]
Mint	*Mentha* spp.	Carvone, limonene, 1, 8-cineole	[176]
Mustard	*Sinapis alba*	Allyl isothiocyanate, phenethyl isothiocyanate	[177]
Nutmeg	*Myristica fragrans*	Eugenol, methyleugenol, methylisoeugenol, elemicin, myristicin, safrole	[178]
Onion	*Allium cepa*	Quercetin, allyl propyl disulphide, protocatechuic acid, quercetin dimer, quercetin trimer, quercetin 4-*o*-β-glucoside, quercetin 3,4-*o*-β-diglucosides	[54, 179]
Parsley	*Petroselinum crispum*	Apiole, apigenin, *p*-1,3,8-menthatriene, β-phellandrene, myrcene, rutin, myristicin	[180]
Red pepper	*Capsicum*	Capsaicin, β-carotene, zeaxanthin, lutein, caffeic acid, capsanthin	[55]
Rosemary	*Rosmarinus officinalis*	Ursolic acid, carnosol, rosmarinic acid, carnosic acid, α-pinene, camphor, limonene, camphene, borneol, cineole, (Z)-linalool oxide, bornyl acetate	[62]
Saffron	*Crocus sativus*	Safranal, picrocrocin, crocetin, crocin	[181]
Sage	*Salvia officinalis*	1,8-cineole, camphor, α-thujone, β-thujone, viridiflorol, borneol	[182]
Sesame	*Sesamum indicum*	Sesamin, sesamolin, sesamol, sesamolinol, γ-tocopherol, phytic acid, linoleic acid, oleic acid, β-sitosterol, campesterol, stigmasterol, Δ5-avenasterol, palmitic acid, stearic acid	[183]
Star anise	*Illicium verum*	Estragole, aretrans-anethole, limonene, phenylpropanoids	[64]
Thyme	*Thymus vulgaris*	Thymol, carvacrol, *p*-cymene, gamma-terpinene, linalool, borneol, β-caryophyllene, carvacrol methyl ether, caryophyllene oxide	[184]
Turmeric	*Curcuma longa*	Curcumin (diferuloylmethane), demethoxycurcumin, bisdemethoxycurcumin	[48]
Vanilla	*Vanilla planifolia*	Vanillin, ethyl vanillin, vanillyl alcohol, vanillic acid, *p*-coumaric acid, ferulic acid, 4-hydroxybenzyl alcohol, 3, 4-dihydroxybenzaldehyde, 4-hydroxybenzoic acid, 4-hydroxybenzaldehyde, piperonal	[185]

## Active components of spices, inflammatory pathways, and chronic diseases

Increasing lines of evidence have established the efficacy of the principal components of spices in preventing as well as alleviating different types of chronic diseases. The main components of spices and their curative potentials are discussed below:

### 1,8-Cineole

**Fig. 3:**
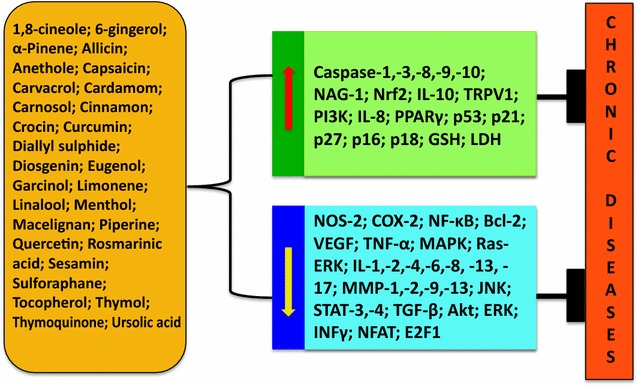
Different bioactive components of spices and their molecular molecular mechanisms against different chronic diseases

1,8-Cineole (Cin) is a monoterpene oxide found in variety of spices such as basil, cardamom, and sage [4]. Cin has been used to treat multiple inflammatory disorders such as bronchitis, sinusitis, chronic rhinitis, and asthma (Table 2). Cin has been shown to downregulate NOS-2, COX-2, and NF-κB, hence showing its potential as an anti-inflammatory agent [60]. Moreover, Cin also attenuated the colonic damage in trinitrobenzene sulfonic acid (TNBS)-induced colitis in rats; decreased acute pulmonary inflammation in vivo; ameliorated acute pancreatitis in vivo via downregulation of cytokines, oxidative stress and NF-κB [38, 65, 66]. In AD, insoluble amyloid β deposits induced inflammation. However, it has been found that 1,8-cineole significantly lowered the expression of proinflammatory cytokines TNF-α, IL-1β and IL-6 in amyloid β toxicated PC12 cells [67]. In addition, numerous studies also showed its potential in preventing different chronic diseases such as asthma, colitis, COPD, pancreatitis, etc. by modulation of inflammatory pathways including TNF-α, COX-2, NF-κB, IL-1β, etc. [66–69] (Table 2) (Fig. 3).

**Table 2:** Spice derived compounds and their mechanism of actions against different chronic diseases

Compound	Chronic diseases	Mechanism of action	References
1,8-cineole	Alzheimer’s disease	↓NOS-2, ↓COX-2, ↓NF-κB	[67]
	Bronchial asthma	↓PGE2, ↓LTB4	[186]
	Colitis	↓Myeloperoxidase	[38]
	COPD	–	[69]
	Pancreatitis	↓NF-κB	[66]
	Ulceration	↓Myeloperoxidase	[38]
6-gingerol	Allergic rhinitis	↓T cell activity	[75]
	Alzheimer’s disease	↑Nrf2	[72]
	Colorectal cancer	↑NAG-1	[70]
	Diabetes	↓VEGF	[71]
	Osteoporosis	↓TNF-α	[187]
	Steatohepatitis	↓NF-κB, ↓TNF-α, ↓IL-6	[73]
α-Pinene	Acute pancreatitis	↓TNF-α, ↓IL-1β, ↓IL-6	[78]
	Arthritis	↓JNK, ↓iNOS, ↓MMP-1, ↓MMP-13	[188]
	Rhinitis	↓IKK-β, ↓Caspase-1	[76]
Allicin	Ankylosing spondylitis	↓IL-6, ↓IL-8, ↓TNF-α	[189]
	Alzheimer’s disease	↑Nrf2	[190]
	Chronic kidney disease	↑Nrf2	[191]
	Gastric cancer	↑G2/M arrest, ↑ER stress	[192]
	Glioblastoma multiforme	↓ERK	[193]
	Hypercholesterolemia	↓TNF-α, ↓NF-κB	[194]
	Recurrent aphthous ulcer	↓TNF-α	[195]
	Type 1 diabetes	–	[196]
	Ulcerative colitis	↓IL-6, ↓STAT3	[18]
Anethole	Breast cancer	↓NF-κB	[197]
	Bronchial dysplasia	–	[198]
Capsaicin	Atherosclerosis	↑TRPV1	[199]
	Alzheimer’s disease	↑Synapsin I; ↑PSD93	[112]
	Bladder cancer	↓FOXO3a	[110]
	Cholangiocarcinoma	↑PI3K/Akt/mTOR	[200]
	Colon cancer	↑Caspase-8, -9, -3	[201]
	Gastrointestinal disorders	–	[202]
	Lung cancer	↓E2F	[114]
	Cardiac hypertrophy and fibrosis	↑TRPV1	[199]
	Pancreatitis	↓ERK, ↓c-Jun, ↓Hedgehog	[203]
	Prostate cancer	↓p27	[113]
Carvacrol	Arthritis	↓Myeloperoxidase	[204]
	Asthma	↓IL-4, ↓TGF-β, ↓IL-17	[205]
	Atherosclerosis	↓MAPK	[206]
	Colon cancer	↓iNOS, ↓IL-1β	[207]
	COPD	↑IL-8	[31]
	Gastric ulcers	↓Prostanoids	[208]
	Intestinal mucositis	↑TRPA1 receptor	[209]
	Pancreatitis	↓AST, ↓ALT, ↓LDH	[210]
	Periodontitis	↓Myeloperoxidase	[211]
Cardamom	Colon cancer	↓COX-2, ↓iNOS	[212]
	Forestomach cancer	↑GSH, ↓LDH	[213]
Carnosol	Brain damage by chronic stress	↑MDA	[214]
	Colon cancer	–	[215]
	Lymphoma	–	[215]
Cinnamon	Arthritis	↓IL-2,-4, ↓IFNγ	[120]
	Alzheimer’s disease	↑p21rac	[121]
	Colitis	↓COX-2	[216]
	Diabetes	↓AP-1	[217]
	Hyperglycemia	↑PPARγ	[218]
	Inflammatory disorders	↓p38, ↓JNK, ↓ERK1/2, ↓STAT4	[219]
	Melanoma	↓AP-1	[217]
	Multiple sclerosis	↑Tregs	[119]
	Parkinson’s disease	↓ Aβ polypeptide	[122]
Coriander	Alzheimer’s disease	↓Aβ42-induced ROS, ↓ERK	[220, 221]
	Atherosclerosis	–	[222]
	Colitis	–	[223]
	Dermatitis	↓IgE, ↓TNF-α, ↓INFγ, ↓IL-1,-4,-13	[224]
	Diabetes	↑Insulin release	[225]
	Rheumatism	–	[226]
Crocin	Alzheimer’s disease	↓Aβ peptide	[227]
	Asthma	↓p-ERK, ↓p-JNK, ↓p-p38	[228]
	Colitis	↓INFγ, ↓COX-2	[16]
	Diabetes	↓TNF-α, ↓IL-1β	[229]
	Liver cancer	↓NF-κB, ↓TNF-α, ↓IL-6, -10	[230]
	Rheumatoid arthritis	↓iNOS, ↓TNF-α, ↓IL-1β, -6	[231]
Curcumin	Alzheimer’s disease	↑PI3K, ↑Akt	[87]
	Asthma	↑Nrf2/HO-1	[89]
	Atherosclerosis	↓IL-1β, -6, ↓TNF-α, ↑PPARγ	[232]
	Cancer	↓Multiple pathways	[160, 161]
	Chagas myocarditis	↓NFAT/COX-2/PGE2	[233]
	COPD	↓p66Shc	[234]
	Colitis	↓STAT3	[235]
	Diabetes	↓NF-κB, ↓NO	[236]
	Epilepsy	↓IL-1β, ↓IL-6, ↓TNF-α	[237]
	Gastric ulcer	↓Acetylation of histone H3	[238]
	Hepatitis	↓PGC-1α	[239]
	Irritable bowel disease	↓p38 MAPK, ↓IL-1β, -10	[240]
	Lupus nephritis	↓IgG1, ↓IgG2a	[241]
	Oral lichen planus	–	[240]
	Psoriasis	↓TNF-α, ↓IFN-γ, ↓IL-2, -12, -22,	[242]
	Prostatitis	↓IL-8, ↓TNF-α	[88]
	Ulcerative proctitis	–	[240]
	Uveitis	–	[240]
Diallyl sulphide	Asthma	↑Nrf2	[79]
	Colon cancer	–	[82]
	Prostate cancer	↑Caspases-3,-9,-10, ↓Bcl-2	[84]
	Osteoarthritis	↓MMP-1,-3,-13, ↓IL-1β	[81]
	Skin cancer	↑Apoptosis	[83]
Diosgenin	Alzheimer’s disease	↑1,25D3-MARRS	[243]
	Breast cancer	↓Vav2	[93]
	Chronic myeloid leukemia	↓PI3K/Akt/mTOR	[94]
	Diabetes	–	[244, 245]
	Graves’ disease	↓IGF-1, ↓NF-κB, ↓cyclin D1, ↓PCNA	[246]
	Hepatitis C	↓STAT3	[96]
	Liver cancer	↑Caspase-3, -8,-9	[97]
	Osteoarthritis	↓IL-1β	[95]
	Osteoporosis	↓RANKL, ↑OPG	[247]
	Prostate cancer	↓PI3K/Akt/mTOR	[98]
Eugenol	Asthma	↓NF-κB	[101]
	Atherosclerosis	↓ALP, ↓LDH, ↓HMG-CoA	[248]
	Breast cancer	↓E2F1/survivin	[103]
	Cervical cancer	↓Bcl-2, ↓COX-2, ↓IL-1β	[102]
	Depression	↑MTT-III	[249]
	Diabetes	↓AST, ↓ALT, ↓LDH, ↓ALP	[100]
	Gastric cancer	↓NF-κB	[104]
	Hepatic steatosis and fibrosis	↓SREBP1	[250]
	Hyperglycemia	↓Glycogen phosphorylase b	[251]
	Skin cancer	↓NF-κB, ↓iNOS, ↓IL-6, ↓TNF-α, ↓PGE2	[252]
Garcinol	Allergy	↓STAT3	[106]
	Breast cancer	↓Caspase-3, ↓NF-κB	[125]
	Cardiovascular diseases	↓STAT3	[106]
	Colon cancer	↓PK 1/2, PI3K/Akt/p70 ribosomal S6 kinase	[123]
	Diabetes	↓STAT3	[106]
	Head and neck cancer	↓STAT3, ↓NF-κB	[126]
	Lung cancer	↓p38-MAPK	[127]
	Oral squamous cell carcinoma	↓NF-κB	[116]
	Pancreatic cancer	↓Wnt/β-catenin, ↓miR-200s	[128]
	Prostate cancer	↑mTOR, ↑Akt	[253]
Limonene	Asthma	↓IL-5, -13, ↓MCP-1	[254]
	Breast cancer	–	[255]
	Colitis	↓NF-κB	[256]
	Colorectal cancer	–	[255]
	Skin cancer	↓Ras-ERK	[257]
Linalool	Diabetes	↓TGF-β1	[258]
	Skin cancer	↓IL-6, ↓COX-2, ↓VEGF, ↓Bcl-2	[259]
	Leukemia	↑p53, ↑p21, ↑p27, ↑p16, ↑p18	[260]
	Cervical cancer	↑p53, ↑p21, ↑p27, ↑p16, ↑p18	[260]
	Colon cancer	↑Hydroxy radical	[261]
Menthol	Pancreatic cancer	↓Focal-adhesion kinase	[262]
	Depression	↑IL-1β,-6, ↑TNF-α	[263]
	Skin cancer	↓NF-κB, ↓ERK, ↓p38	[264]
	Napkin dermatitis	–	[265]
	Neuropathic pain	↑TRPM8	[266]
Macelignan	Alzheimer’s disease	–	[267]
	Asthma	↓IL-4, ↓GATA3	[268]
	Type 1 allergy	↓Akt, ↓TNF-α, ↓MAPK, ↓c-Jun	[269]
Piperine	Alzheimer’s disease	–	[270]
	Arthritis	↑IL-10	[151]
	Asthma	↓IL-4, -5, ↓NF-κB	[150]
	Breast cancer	↑p53, ↓MMP-9,-2, ↓c-Myc, ↓VEGF	[271]
	Chronic gastritis	↓IL-1β, ↓IFN-γ, ↓IL-6, ↓iNOS	[272]
	Colorectal cancer	–	[273]
	Depression	↑BDNF	[274]
	Endometritis	↓NF-κB, ↓MAPK	[148]
	Fibrosarcoma	↓MMP-9	[275]
	Gastric cancer	↓STAT3	[154]
	Parkinson’s disease	↓IL-1β, ↓TNF-α	[276]
	Triple negative breast cancer	↓Survivin, ↓p65	[277]
	Ulcerative colitis	–	[278]
Quercetin	Arthritis	↓NF-κB, ↓1β, ↓MCP	[139]
	Atherosclerosis	↑Akt	[147]
	Atopic dermatitis	↓JAK-STAT	[142]
	Breast cancer	↓Twist	[140]
	Diabetes mellitus	–	[143]
	Hepatitis	↑Nrf2	[138]
	Inflammatory bowel disease	↑GSH	[141]
	Periodontitis	↓IL-1β, ↓TNF-α, ↓RANKL, ↓iCAM-1	[279]
	Psoriasis	–	[144]
Rosmarinic acid	Asthma	↓ERK, ↓JNK, ↓p38MAPK	[19]
	Amyotrophic lateral sclerosis	↓HNE	[280]
	Colitis	↓NF-κB, ↓STAT3	[281]
	Colorectal cancer	↓IL-6/STAT3	[282]
	Gastric cancer	↓IL-6/STAT3	[283]
	Hepatocellular carcinoma	↓NF-κB	[284]
	Leukemia	–	[285]
	Neuropathic pain	↓COX-2, ↓PGE2, ↓IL-1β, ↓MMP-2	[286]
	Osteoporosis	↓NFATc1	[287]
	Pancreatitis	↓NF-κB	[288]
	Psoriasis	↓IL-1β, ↓IL-6, -8, ↓CCL20, ↓TNF-α	[289]
	Rhinoconjunctivitis	↓iCAM-1, ↓VCAM-1, ↓COX-2, ↓MIP-2	[290]
Sesamin	Asthma	↓IκB-α, ↓NF-κB	[291]
	Atherosclerosis	↓MCP-1, ↓IL-1α, ↓IL-6, ↓CXCL-16	[292]
	Breast cancer	↓VEGF, ↓MMP-9	[293]
	Diabetes	↓FBS, ↓HbA1C, ↓TNF-α	[294]
	Gall bladder carcinoma	↓NF-κB-IL-6-Stat3-Twist	[295]
	Osteoarthritis	↑Nrf2	[296]
	Prostate cancer	↓p38-MAPK, ↓NF-κB	[297]
Sulforaphane	Alzheimer disease	↑NLRP3	[298]
	Atherosclerosis	–	[299]
	Breast cancer	↓Bcl-2, ↑Caspase-3,-9	[158]
	Cardiovascular diseases	↑Nrf2	[155]
	Colorectal cancer	↑AP-1	[158]
	Diabetes	↓RAGE	[157]
	Lung cancer	↓Bcl-2, ↑Caspase-3, ↑Bax	[158]
	Multiple sclerosis	↑Nrf2	[159]
Tocopherol	Atherosclerosis	↓IL-6,-10, ↓MCP-1, ↓TNF-α	[300]
	Colitis	↓IL-6	[301]
	Colon cancer	↓8-HDOG, ↓γ-H2AX	[302]
	Lung cancer	↓8-HDOG, ↓γ-H2AX	[302]
	Mammary hyperplasia	↓PCNA, ↓COX-2, ↑PPARγ, ↑Nrf2	[303]
Thymol	Asthma	↓NF-κB	[304]
	Endometritis	↓TNF-α, ↑IL-1β, ↑iNOS, ↑COX-2	[305]
	Gastric ulcer	↑ PGEs, ↑ATP K(+) channels	[306]
	Mastitis	↓IκBα, ↓NF-κB, ↓ERK, ↓JNK	[307]
Thymoquinone	Allergic conjunctivitis	↓Eosinophils, ↓IgE, ↓histamine	[133]
	Asthma	↓CD31, ↓α-SMA	[131]
	Bladder cancer	↓NF-κB, ↓XIAP	[134]
	Cholangiocarcinoma	↓PI3K/Akt, ↓NF-κB	[308]
	Depression	↓TBARS, ↑GSH	[309]
	Diabetes mellitus	↓p44/42, ↓p38-MAPKs	[310]
	Gastric cancer	↓STAT3, ↓JAK2, ↓c-Src	[137]
	Lung cancer	↓PCNA, ↓CD1, ↓MMP-2, ↓ERK1/2	[135]
	Multiple myeloma	↓Ki-67, ↓VEGF, ↓Bcl-2, ↓p65	[311]
	Myeloid leukemia	↓NF-κB, ↓CD1, ↓COX-2, ↓MMP-9	[312]
	Osteoarthritis	↓IL-1β-induced MMP-1,-3,-13	[130]
	Ovarian cancer	↑pH2AX, ↓NF-κB	[136]
	Rheumatoid arthritis	↓ASK1	[132, 313]
	Rhinosinusitis	–	[314]
Ursolic acid	Asthma	↓IL-5, -13	[315]
	Colitis	↓NF-κB	[316]
	Prostate cancer	↑Caspase-3,-9, ↓ROCK/PTEN	[317]
	Rheumatoid arthritis	↓PGE2	[318]

### 6-Gingerol

6-Gingerol, the main active component of ginger, is shown to possess different biological activities such as anti-oxidative, anti-inflammatory and anti-proliferative properties [51]. Its therapeutic effect was observed against various chronic diseases such as AD, colorectal cancer and diabetes [70–72] (Table 2) (Fig. 3). For example, 6-Gingerol can induce downregulation of inflammatory cytokines such as monocyte chemoattractant protein-1 (MCP-1), TNF-α, and IL-6, and NF-κB thereby, ameliorating steatohepatitis in vivo [73]. 6-gingerol also has a protective role against colitis in vivo through the activation of adenosine monophosphate-activated protein kinase (AMPK) pathway [74]. Studies have shown that this nutraceutical is a potential candidate for the treatment of diabetes. Diabetic rat treated with a ginger extract containing 5% of 6-gingerol significantly attenuated the expression of NF-κB and inhibited the activity of TNF-α and VEGF [71]. Moreover, 6-gingerol possesses anti-tumorigenic and pro-apoptotic properties. For instance, 6-gingerol promoted cell apoptosis in human colorectal cancer cells via the upregulation of nonsteroidal anti-inflammatory drug (NSAID)-activated gene-1 (NAG-1) [70]. Another study also demonstrated that 6-gingerol suppressed cytokine production for T cell activation and proliferation, hindering B cell and mast cell activation, thereby alleviating symptoms of allergic rhinitis (AR) [75].

### α-Pinene

α-Pinene is a monoterpene, found mainly in eucalyptus oils and oils of aromatic plants such as rosemary. It is known to possess antimicrobial, apoptotic, antimetastatic, and antibiotic properties [76]. α-pinene is one promising agent for treatment of various inflammatory diseases as it has been found to suppress MAPKs and NF-κB pathway [77] (Fig. 3). The inflammation associated with acute pancreatitis is considerably reduced by treatment with α-pinene in vivo via the downregulation of TNF-α, IL-1β, and IL-6 [78]. Furthermore, treatment of AR mouse model with α-pinene significantly inhibited receptor-interacting protein 2 (RIP2), IκB kinase (IKK)-β, NF-κB, and caspase-1, thereby making α-pinene an anti-allergic agent against AR [76].

### Diallyl sulphide (DAS)

Diallyl sulphide (DAS) is the major organo sulphur compound of garlic. It is a potential agent for treatment of airway inflammation such as asthma through its ability to regulate nuclear factor-E2-related factor 2/haemoxygenase-1 (Nrf2/HO-1) and NF-κB pathway [40]. Likewise, in vivo studies have also shown that DAS alleviated ovalbumin (OVA)-induced allergic asthma by inhibiting inflammatory factors such as ROS, NF-κB and 8-hydroxy-2′-deoxyguanosine, 8-iso-prostaglandin F2α, and increasing the activation of Nrf2 [79]. In case of osteoarthritis, DAS was reported to inhibit the expression of COX-2 potentially via NF-κB pathway [80]. In vivo study confirmed that DAS protected the cartilage in the development of osteoarthritis by inhibiting the expression of MMP-1, MMP-3, MMP-13, and IL-1β as well as enhancing the production of collagen II [81]. DAS has also been demonstrated to have anticancer properties against different cancers such as colon cancer, prostate cancer, skin cancer, etc. via modulation of inflammatory pathways [82–84].

### Curcumin

Curcumin, an active component of turmeric, is the most widely studied nutraceutical. It is known to possess anti-antioxidant, anti-bacterial, anti-cancer, , anti-fungal, anti-inflammatory and anti-viral activities. Thus, it is a potential agent against various chronic illnesses. It has been shown to modulate various inflammatory mediators including IL-6, TNF-α, PI3K/Akt, STAT3, IL-27, NF-κB, MAPK, etc. in various preclinical and clinical studies (Table 2) (Fig. 3). For example, inflammation of microglia cells prompts central nervous system (CNS) disorders. Interestingly, curcumin attenuates PI3K/Akt phosphorylation, NF-κB activation, and iNOS in lipopolysaccharide (LPS)-induced inflammatory responses in microglial cells [85]. This nutraceutical also effectively reduced the inflammatory responses in mastitis mice model via suppression of TLR4-mediated NF-κB signaling pathway [86]. Furthermore, curcumin was shown to ameliorate the insulin signaling in the brain of AD in vivo, thus showing its feasibility for treatment of AD [87]. Additionally, curcumin also alleviated chronic nonbacterial prostatitis by downregulating TNF-α, IL-6, and IL-8 in vivo [88]. Furthermore, it has been demonstrated that curcumin reduced asthmatic airway inflammation by activating Nrf2/HO-1 signaling pathway [89]. In case of human non-small cell lung cancer, this potent compound induced apoptosis via the upregulation of micro RNA, miR-192-5p and downregulation of PI3K/Akt signaling pathway [90]. Also, this compound was reported as a protectant against severe acute pancreatitis via attenuation of NF-κB in vivo [91]. This compound is known to inhibit cancer cell proliferation, survival, invasion, angiogenesis, metastases, chemoresistance, and radiation resistance in different types of cancers via modulation of different signaling pathways including NF-κB. Approximately, over 120 clinical trials have proven its potential to treat different chronic diseases without showing any adverse side effects. Curcumin has been shown to inhibit IBD, colitis, rhinitis, oral lichen planus, psoriasis, and prostatitis in various clinical trials. It has also been shown to inhibit cancer alone or in combination with standard chemotherapeutic agents in many clinical trials. So far, curcumin is the most extensively studied spice derived component for the treatment of different chronic diseases in both preclinical and clinical settings.

### Diosgenin

Diosgenin is a bioactive compound obtained from the spice *Trigonella foenum*-*graecum* L. (fenugreek). Over the years, this spice has been known for its anti-carcinogenic, anti-diabetic, anti-oxidant, hypocholesterolemic and immunological properties. Because of its anti-inflammatory activities, diosgenin is a potential agent for various chronic diseases including AD, breast cancer, chronic myeloid leukemia, and osteoarthritis [92–95] (Table 2) (Fig. 3). For instance, it has been shown to inhibit the expression of MMP-3, MMP-13, iNOS, and COX-2 on human osteoarthritis (OA) in vivo, thus, making diosgenin a suitable agent for OA therapy [95]. Additionally, diosgenin was found to exhibit anti-viral activity against hepatitis C in vitro; induce apoptosis in hepatocellular carcinoma and prostate cancer and inhibit migration of human breast cancer in vitro [93, 96–98]. Diosgenin also enhanced ROS-dependent autophagy and cytotoxicity in chronic myeloid leukemia cells via inhibition of mammalian target of rapamycin (mTOR) signaling pathway [94]. This compound was also reported to prevent bone loss on retinoic acid-induced osteoporosis in vivo [99].

### Capsaicin

Aforementioned, capsaicin (trans-8-methyl-*N*-vanillyl-6-nonenamide) is a principal component of the spice red pepper (*Capsicum*) [100, 101]. It is highly efficacious in ameliorating several chronic diseases such as asthma, diabetes, cancers of breast, cervical, stomach, etc. via the inhibition of STAT3, NF-κB, PGE2, IL-6, TNF-α, etc. [102–107] (Table 2) (Fig. 3). Additionally, capsaicin also exhibits anticancer activity against cancer of the colon, lung, prostate, skin and tongue [46]. Studies revealed that capsaicin inhibits inflammatory cytokines such as IL-1β, IL-6, and TNF-α by upregulating Liver X receptor α (LXRα) [108]. Capsaicin can also reduce inflammation in salivary glands via inhibition of NF-κB pathway [109]. This efficient compound also effectively induced cell cycle arrest in bladder cancer cells via forehead box O3a (FOXO3a)-mediated pathway [110]. In vitro and in vivo studies also revealed that capsaicin ameliorated chronic diseases such as AD, skin inflammation, small cell lung cancer, etc. [111–114].

### Eugenol

Eugenol, the active principle from clove extract, is well known for its anti-inflammatory properties via modulation of inflammatory biomarkers such as TNF-α, IL-1, IL-6, COX-2, PGE2, NF-κB, etc. [115] (Table 2) (Fig. 3). In addition, it has been shown to inhibit various chronic diseases in preclinical studies (Table 2). For instance, eugenol was shown to restrict the progression of asthma in vivo by inhibition of NF-κB pathway [101]. This compound also inhibited cell proliferation in gastric cancer in vivo by suppressing NF-κB pathway [104]. Eugenol was found to enhance the efficacy of anti-cancer drug, gemcitabine and exert anti-inflammatory activity in human cervical cancer cells [102]. In addition, eugenol was shown to inhibit skin cancer via attenuation of c-Myc, H-ras and induction of p53 dependent apoptosis and induction of apoptosis in breast cancer cells via E2F1/survivin downregulation [103, 116]. Numerous investigations further revealed that eugenol exhibits anti-depressant as well as anti-diabetic activities [100, 117].

### Cinnamaldehyde

Cinnamaldehyde (CM) is the active component of the spice cinnamon (*Cinnamomum zeylanicum*). This component is widely known for its anti-inflammatory, anti-microbial, anti-oxidant, anti-tumor, cholesterol lowering and immunomodulatory properties [57]. CM exerted its anti-inflammatory effect in gastric inflammation by inhibiting NF-κB activation [118]. Cinnamon can also reduce allergic encephalomyelitis in vivo via regulatory T cells [119]. Cinnamon bark has a prominent action in reducing inflammation in arthritis model in vivo via inhibiting cytokines such as IL-2, IL-4, and interferon γ (IFNγ), hence may be regarded as a potent anti-rheumatic agent [120]. Moreover, cinnamon is also effective for the treatment of neurodegenerative diseases such as AD [121, 122] (Table 2).

### Garcinol

Garcinol is a polyisoprenylated benzophenone isolated from the plant *Garcinia indica* (Kokum) [106]. A functional investigation has revealed the anti-carcinogenic, anti-inflammatory and anti-oxidative properties of garcinol [123]. Studies showed that garcinol inhibited the proliferation of breast cancer cells in vitro [124]. Additionally, it also sensitized breast cancer cells to a chemotherapeutic agent, taxol via downregulation of NF-κB/Twist1 and caspase-3/iPLA(2) signaling pathways in a mouse 4T1 breast tumor model [125]. This active component also inhibited inflammation-associated colon carcinogenesis in vivo [123]. Furthermore, garcinol also mediated anti-tumor effect by inhibiting the constitutive activation of STAT3 and NF-κB in squamous cell carcinoma of the head and neck [126]. It has also been reported that garcinol exerted its anti-cancer activity by inducing downregulation of p38-MAPK signaling in lung cancer; NF-κB inhibition in oral cancer; modulation of epithelial–mesenchymal transition (EMT) and Wnt signaling in breast cancer [105, 127, 128].

### Thymoquinone

Thymoquinone is isolated from black cumin (*Nigella sativa)*. It has been shown to possess anti-inflammatory, anti-oxidant, and chemopreventive activities [129]. A recent report has depicted that this bioactive component inhibited IL-1β-induced inflammation via downregulating NF-κB and MAPKs signaling in human osteoarthritis chondrocytes [130]. It also prevented inflammation, neoangiogenesis, and vascular remodeling in asthma in vivo [131]. Thymoquinone also inhibited TNF-α-induced inflammation and cell adhesion in RA, thus making it a promising anti-inflammatory agent [132]. Studies also reported the ameliorative activity of thymoquinone against ovalbumin-induced allergic conjunctivitis in vivo [133]. Additionally, it was also found to be effective against cancer of the bladder, lung, ovarian, gastric, etc. Thymoquinone portrayed its anti-tumor function via inactivation of PI3K/Akt, ERK, NF-κB and STAT3 pathways [134–137] (Table 2) (Fig. 3).

### Quercetin

Quercetin is a dietary flavonoid obtained from onions. The anti-cancer, anti-inflammatory, and anti-oxidant properties of this phytochemical are demonstrated by numerous studies. Quercetin is effective against various chronic diseases including arthritis, breast cancer, dermatitis, diabetes, IBD, hepatitis, psoriasis, etc. due to its ability to inhibit the dysregulated inflammatory pathways involved in these chronic diseases (Table 2) [138–144]. The anti-inflammatory properties of quercetin is attributed to its ability to downregulate NF-κB and MAPK pathways and enhance PI3K/Akt and Nrf2 pathways [145–147] (Table 2) (Fig. 3).

### Piperine

Piperine is the principal plant alkaloid isolated from black pepper (*Piper nigrum*) and long pepper (*Piper longum*). Piperine has several biological properties including analgesic, anti-convulsant, anti-tumor and anti-inflammatory activities [148]. Several studies have shown that piperine could attenuate the inflammatory response associated with chronic diseases such as AD, asthma, arthritis, chronic gastritis, endometritis, Parkinson’s disease, etc. [149–151] (Table 2). The anti-inflammatory activity of piperine in these chronic diseases is achieved via downregulation of inflammatory pathways such as NF-κB, MAPK, AP-1, COX-2, NOS-2, IL-1β, TNF-α, PGE2, STAT3, etc. [148, 149, 151–154] (Table 2) (Fig. 3).

### Sulforaphane

Sulforaphane is an isothiocyanate (sulphur containing compounds) distributed amongst cruciferous vegetables including mustard. Studies have shown that sulphoraphane possesses anti-cancer and cardioprotective activities [155]. It elicits protection against cardiovascular diseases via activation of Nrf2 [155]. Studies also reported that sulforaphane represents a promising agent for treatment of chronic diseases such as AD, bladder cancer, colorectal cancer, diabetes, and  lung cancer [156–158] (Table 2). Another study has also suggested that sulforaphane inhibit pro-inflammatory signaling through inhibition of NF-κB pathway [159] (Fig. 3).

**Fig. 4:**
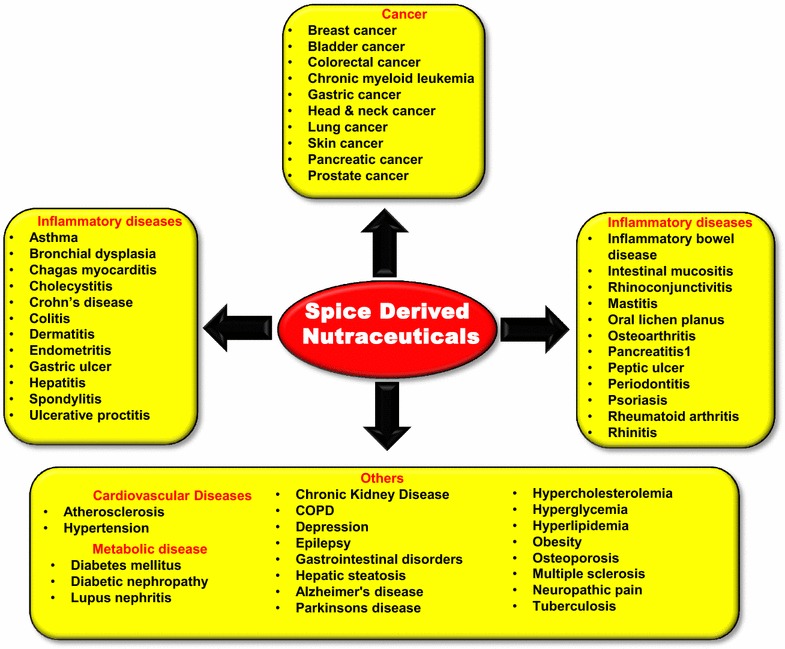
Spice derived nutraceuticals against various chronic diseases

Besides these active components, other compounds found in spices includes allicin (garlic), anethole (fennel), carnosol (rosemary); linalool (coriander), crocin (saffron), sesamin (sesame seed), ursolic acid (basil), carvone (mint), myristicin (nutmeg), etc. These potent ingredients of diverse spices have been found to aid in preventing and alleviating various chronic diseases (Fig. 4), mostly by downregulating signaling pathways such as NF-κB, STAT3 and ERK/MAPK pathways [129, 146, 148, 159–163].

## Conclusion

Overall, it is evident from these studies that the allure of spices is attributed not only to their aroma, but also more importantly, to their wellness power. The spice-derived compounds can interact with multiple targets and alter the dysregulated inflammatory pathways and mediators associated with chronic diseases. Hence, with the fatal side effects and inflating cost of modern therapeutics, spices and their active components hold a huge guarantee for the development of affordable, novel and safe drugs against chronic diseases. However, in-depth scientific investigations are required to completely determine the potential of the spice-derived nutraceuticals and open new avenues for the better management of patients with chronic diseases.

## Abbreviations

1,25D3-MARRS: 1,25D3-membrane-associated, rapid response steroid-binding protein
ALP: alkaline phosphatase
ALT: alanine aminotransaminase
AP-1: activator protein 1
ASK1: apoptosis signal-regulating kinase 1
AST: aspartate transaminase
ATP: adenosine triphosphate
Aβ: amyloid beta
BDNF: brain-derived neurotrophic factor
CAM-1: cell adhesion molecule-1
CCL20: chemokine (C–C motif) ligand 20
CD1: cyclin D1
COX-2: cyclooxygenase-2
FBS: fasting blood sugar
FOXO: Forkhead box-O
GSH: glutathione
HbA1c: glycated haemoglobin
HDOG: 8-hydroxydeoxyguanosine
HNE: 4-hydroxy-2-nonenal
HMG-CoA: 3-hydroxy-3-methyl-glutaryl-CoA reductase
HO-1: heme oxygenase-1
iCAM-1: intercellular cell adhesion molecule-1
IGF-1: insulin-like growth factor 1
IgG: immunoglobulin G
INF-γ: interferon-γ
iNOS: inducible nitric oxide synthase
IκB kinase β: I kappa B kinase beta
IκBα: inhibitory factor kappa B alpha
JAK2: Janus kinase 2
JNK: c-JUN N-terminal kinase
LDH: lactate dehydrogenase
LTB4: leukotriene B4
MAPK: mitogen-activated protein kinases
MCP-1: monocyte chemoattractant protein-1
MDA: malondialdehyde
MIP: macrophage inflammatory protein
MTT-III: metallothionein-III
NAG-1: nonsteroidal anti-inflammatory drug (NSAID)-activated gene-1
NF-κB: nuclear factor kappa B
NFAT: nuclear factor of activated T-cells
NFATc1: nuclear factor of activated T cells cytoplasmic 1
NLRP3: nucleotide-binding oligomerization domain-like receptor family, pyrin domain-containing-3
NO: nitric oxide
NOS: nitric oxide synthases
Nrf2: nuclear factor erythroid 2–related factor 2
OPG: osteoprotegerin
PCNA: proliferating cell nuclear antigen
PGC-1α: peroxisome proliferator-activated receptor gamma coactivator 1-alpha
PGE2: prostaglandin E2
PI3K: phosphatidylinositol-3 kinase
PK: protein kinase
PPAR: peroxisome proliferator-activated receptor
PSD93: postsynaptic density protein 93
PTEN: phosphatase and tensin homolog
RAGE: receptor for advanced glycation end products
RANKL: receptor activator of nuclear factor kappa-B ligand
ROCK: rho-associated protein kinase
SREBP-1: sterol regulatory element-binding protein-1
STAT: signal transducer and activator of transcription
TBARS: thiobarbituric acid reactive substance
TGF-β: transforming growth factor beta
TH2: T-helper 2
TLR4: Toll-like receptor 4
TNF-α: tumor necrosis factor-alpha
Tregs: regulatory T cells
TRPA1: transient receptor potential cation channel, subfamily A, member 1
TRPM8: transient receptor potential cation channel subfamily M member 8
TRPV1: transient receptor potential vanilloid type 1
VCAM: vascular cell adhesion molecule
α-SMA: alpha-smooth muscle actin
